# Identification of a human immunodominant B-cell epitope within the immunoglobulin A1 protease of *Streptococcus pneumoniae*

**DOI:** 10.1186/1471-2180-7-113

**Published:** 2007-12-18

**Authors:** Francesca De Paolis, Elisa Beghetto, Andrea Spadoni, Francesca Montagnani, Franco Felici, Marco R Oggioni, Nicola Gargano

**Affiliations:** 1 Kenton laboratories, Kenton Srl, Rome, Italy; 2 Department of Molecular Biology, University of Siena, Italy; 3 Department of Microbiology, Genetics and Molecular Biology, University of Messina, Italy

## Abstract

**Background:**

The IgA1 protease of *Streptococcus pneumoniae *is a proteolytic enzyme that specifically cleaves the hinge regions of human IgA1, which dominates most mucosal surfaces and is the major IgA isotype in serum. This protease is expressed in all of the known pneumococcal strains and plays a major role in pathogen's resistance to the host immune response. The present work was focused at identifying the immunodominant regions of pneumococcal IgA1 protease recognized by the human antibody response.

**Results:**

An antigenic sequence corresponding to amino acids 420–457 (epiA) of the *iga *gene product was identified by screening a pneumococcal phage display library with patients' sera. The epiA peptide is conserved in all pneumococci and in two out of three *S. mitis *strains, while it is not present in other oral streptococci so far sequenced. This epitope was specifically recognized by antibodies present in sera from 90% of healthy adults, thus representing an important target of the humoral response to *S. pneumoniae *and *S. mitis *infection. Moreover, sera from 68% of children less than 4 years old reacted with the epiA peptide, indicating that the human immune response against streptococcal antigens occurs during childhood.

**Conclusion:**

The broad and specific recognition of the epiA polypeptide by human sera demonstrate that the pneumococcal IgA1 protease contains an immunodominant B-cell epitope. The use of phage display libraries to identify microbe or disease-specific antigens recognized by human sera is a valuable approach to epitope discovery.

## Background

*Streptococcus pneumoniae *is a human pathogen causing significant morbidity and mortality worldwide. It is a transient member of the normal bacterial flora that colonizes the upper respiratory tract of the host being a major cause of various diseases such as otitis media, pneumonia, sepsis and meningitis. Despite the constant development of therapeutics, antimicrobial drugs and vaccines, pneumococcal infection still causes severe diseases in young children, elderly people and immunocompromised individuals [1,2]. In adults, pneumococcal infection is the most common cause of community-acquired pneumonia and otitis media and, since the introduction of vaccination against *Haemophilus influenzae *(serotype b) and *Neisseria meningitidis *also the most frequent cause of meningitis.

Current immunization strategies focus on the use of *S. pneumoniae *polysaccharides-based vaccines, employing the 23-valent vaccine, which protects humans from two years of age, and the 7-valent toxoid-conjugated vaccine, used in children less than two years old [3,4]. However, the available vaccines have several limitations due to the low immunogenicity of capsular polysaccharides, the high serotype variability and the genomic plasticity of this bacterium. Therefore, in the last decade there has been a great effort in developing improved vaccines to prevent pneumococcal disease.

Several surface-associated proteins, which are well conserved among the different strains of *S. pneumoniae *and thus represent candidates of choice for the development of novel vaccine formulations have been identified and characterized. Among this class of proteins the immunoglobulin A1 (IgA1) protease is a promising candidate since (i) it plays a major role in pathogen's resistance to the host immune response [5,6], and (ii) it is present in all pneumococcal strains and serotypes [7,8]. The importance of IgA protease is underlined by the fact that this host-specific enzyme is conserved in other pathogens of comparable disease and colonising similar niches [9-11].

The IgA1 protease is one of the two to four large zinc metalloproteinase present in the pneumococcal genome [7,12]. The pneumococcal protease is a polypeptide of about 1900 amino acids associated to the bacterium via N-terminal anchoring [7,13-15]. It is a proteolytic enzyme that specifically cleaves human IgA1 antibodies in the hinge region of the immunoglobulin heavy chain [14,15].

Cross-inhibition experiments performed with sera from immunized rabbits have revealed considerable serological diversity of IgA1 proteases from different *S. pneumoniae *strains [16]. Serological analysis indicated that the sequence repeats domain of *S. sanguis *IgA1 protease was immunogenic in rabbits and in humans, although the antibodies recognizing this region did not inhibit enzyme activity [17]. Specific antibodies reacting with IgA1 protease have been detected in sera from patients hospitalized for pneumococcal infection [18] as well as in young children [19], highlighting the immunogenity of pneumococcal IgA1 protease in humans.

The aim of this work was to identify the immunodominant epitopes of pneumococcal IgA1 protease involved in the human antibody response against bacterial infection.

## Results

**Figure 1:**
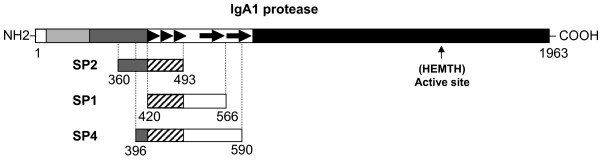
**Schematic representation of the selected phage clones**. Alignment of the selected antigen fragments with the *S. pneumoniae *IgA1 protease of strain R6. The streptococcal IgA1 protease is typically organised in (i) a signal peptide (white box), (ii) a 200 amino acid N-terminal domain with three transmembrane segments and an LPXTG anchor domain (light grey box), (iii) a 200 amino acid fibrillar domain (grey box), (iv) a region with repeat segments possibly involved in the binding of extracellular matrix components (empty box with arrows), and (v) a large central and C-terminal domain containing the active site of the protease (black box). The repeat segments in R6 are three repeats of seventeen amino acids with a conservation of 62 to 75% followed by two repeats of 78 amino acids with 64% identity. The common region of the antigen fragments in shown in diagonal bars.

In a recent study, we isolated several antigenic regions of *S. pneumoniae *proteins by challenging a pneumococcal genome display library with antibodies from one patient infected by the bacterium [20]. In the present work a similar approach was employed by using "ad-hoc" patients' sera which displayed a low titer of antibodies against the pneumococcal antigens identified in the previous study (data not shown). Accordingly, sera from two patients hospitalized for pneumococcal pneumonia were used for a novel screening of the pneumococcal library. Phage pools were analyzed after two round of affinity selection and 30 out of 200 screened phage clones (15%) were isolated by immunoscreening. Among them, 9 clones (30%) contained insert encoding fragments of pneumococcal IgA1 protease and 21 clones (70%) contained insert of other bacterial proteins. At the end of the selection procedure, 3 distinct phage clones whose DNA inserts matched the sequence of the pneumococcal *iga *gene product were identified: clone SP1, with a DNA insert encoding for a protein fragment of 145 amino acids; clone SP2 encoding for a polypeptide of 133 residues; clone SP4 encoding for a polypeptide of 194 amino acids. As shown in Figure 1, all the protein inserts of the selected phages contain a common region of 72 amino acids corresponding to residues 420–493 of pneumococcal IgA1 protease (strain R6).

To characterize the biochemical and immunological properties of the selected protein fragments independently from the phage display context, the DNA inserts of phages SP2 and SP4 were subcloned into vector pGEX-SN [21]. This resulted in the expression of GST fusion proteins which were purified from *E. coli *cells under native conditions by 1-step affinity chromatography. The recombinant proteins were efficiently expressed and purified in large amounts from the cytoplasm of bacterial cells, with the yields of purified GST-SP2 and GST-SP4 being 7 and 5 mg per liter of bacterial culture, respectively. Immunoreactivity of recombinant antigens with immunoglobulins G (IgG) of human sera was examined. To this aim, an enzyme-linked immunoassay was employed (Rec-ELISA). The GST-SP2 and GST-SP4 fusion proteins were assayed with 30 sera collected from healthy adults. As a control, the IgG reactivity against wild-type GST protein was assessed for each serum. The criterion used to assign a specific reactivity against single antigens was an OD_GST-ANTIGEN _greater than twice the OD_GST_. Both the SP2 and SP4 polypeptides specifically reacted with more than 80% of serum samples (data not shown).

**Figure 2:**
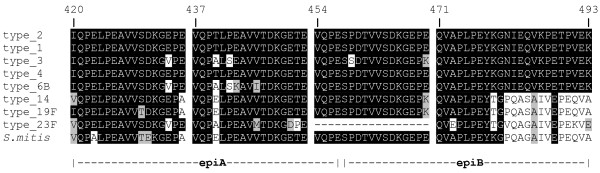
**Comparison of streptococcal IgA1 protease sequences**. The protein region to which peptides epiA and epiB correspond to amino acids 420–493 of the IgA1 protease of *S. pneumoniae *R6. The amino acid sequences are from *S. pneumoniae *type 2 strain R6 (U47687, Q54875), the type 4 strain TIGR4 (NC_003028), and the type 19F strain G54 (AL449925). The sequence of the type 6B strain 670 is from the TIGR Website [35]. BLAST comparisons to further sequences were done on the Sanger Website for the type 1 strain INV104B, the type 3 strain OXC141, the type 14 strain INV200 and the type 23F strain Spanish 23F-1 [36]. The *S. mitis *sequence is from GenBank AAY40355. Identical amino acid sequences are in white letters on a black background.

Next, the presence of immunodominant epitopes within the selected antigen fragments was investigated. To this purpose, the common region of SP2 and SP4 protein fragments, corresponding to amino acids 420–493 of pneumococcal IgA1 protease was analyzed. Figure 2 shows the alignment of this polypeptide of strain D39 to IgA proteases of other pneumococci and related species. In strain D39 such the sequence contains three 17 amino acid repeats which are part of a region of the IgA protease found to show high intraspecies conservation and a high degree of interspecies variability [22]. Pneumococcal IgA proteases fall in this region into two clusters: IgA1 proteases in genomes of strains of serotypes 1, 2, 3, 4 and 6B all have three repeats and a common downstream region while other strains share a common downstream region with *S. mitis *and have from two (type 23F), to seven (type 14), eight (type 19F) or even nine of these highly conserved 17 amino acid repeats. The sequences of IgA proteases of other oral streptococci such as *S. sanguinis *or *S. oralis *can not be aligned in this region since no significant homology was detected.

**Figure 3:**
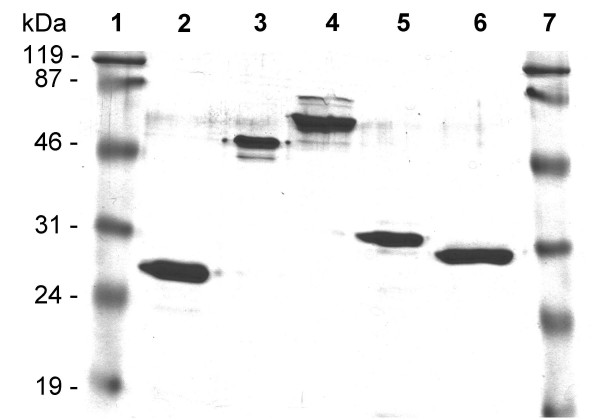
**Characterization of the recombinant fusion proteins**. Purified proteins were subjected to SDS-PAGE analysis and loaded as follows: (1) and (7), molecular weight markers; (2) GST wild-type protein; (3) GST-SP2; (4) GST-SP4; (5) GST-epiA; and (6) GST-epiB.

Two GST fusion proteins, respectively carrying residues 420–457 (epiA) and 458–493 (epiB) of the IgA1 protease from *S. pneumoniae *R6 strain were expressed. As shown in Figure 3, the recombinant GST-epiA and GST-epiB antigens were efficiently purified under native conditions from transformed *E. coli *cells. Immunoreactivity of recombinant antigens with IgG antibodies of human sera was examined and the results were compared to those obtained by use of the largest fragment of the IgA1 protease (clone SP4). The GST-epiA, GST-epiB and GST-SP4 antigens were assayed by Rec-ELISA with serum samples from the two patients hospitalized for pneumococcal pneumonia (used for the library selection) and from 46 healthy individuals. Immunoreactivity of wild-type GST was also assessed for each serum. As shown in Table 1, the recombinant GST-SP4, GST-epiA and GST-epiB proteins specifically reacted with 44 (92%), 43 (89%) and 33 (69%) of the sera, respectively, highlighting a broad antibody recognition of all antigen fragments.

**Table 1:** Immunoreactivity of the *S. pneumoniae *IgA1 protease antigen fragments P^aP^

**Serum no**.	**IgG Rec-ELISA (OD) **P^bP^			
	
	**GST-SP4**	**GST-epiA**	**GST-epiB**	**GST**
PP1	**2.47**	**2.62**	**2.13**	0.26
PP2	**2.19**	**1.63**	**0.45**	0.10
S49	**1.40**	0.48	0.19	0.32
S50	**1.74**	**1.52**	0.23	0.24
S52	**2.75**	**0.57**	0.12	0.21
S53	**0.42**	**0.23**	**0.28**	0.08
S54	**0.32**	**0.18**	0.05	0.05
S58	**1.80**	**0.94**	**0.75**	0.06
S61	**0.15**	**0.42**	**0.23**	0.06
S62	**0.44**	**1.47**	**0.29**	0.13
S66	**0.90**	**0.70**	**0.12**	0.06
S67	0.31	0.39	0.25	0.25
S68	0.13	0.19	0.08	0.11
S69	**1.22**	**0.24**	0.12	0.11
S73	**0.70**	**0.95**	0.13	0.07
S74	**0.44**	**0.42**	**0.34**	0.06
S75	**0.60**	**0.25**	0.13	0.10
S76	**1.64**	**1.72**	**0.43**	0.08
S81	**0.72**	**0.49**	0.14	0.09
S82	0.06	0.07	0.05	0.05
S83	**0.40**	**0.76**	0.21	0.13
S84	**1.01**	**0.73**	**0.16**	0.07
S85	**0.37**	**0.18**	**0.24**	0.08
S87	0.11	0.22	0.15	0.13
S88	**1.27**	**1.15**	**1.47**	0.07
S89	**0.46**	**0.61**	**1.34**	0.15
S90	**2.81**	**2.74**	**0.46**	0.13
S92	**0.90**	**0.90**	**0.44**	0.05
S94	**0.31**	**0.26**	**0.47**	0.09
S96	**0.52**	**0.54**	**0.60**	0.06
S98	**1.09**	**0.58**	**0.12**	0.05
S100	**2.01**	**0.64**	**0.89**	0.06
S101	**1.84**	**0.76**	0.14	0.08
S102	**0.56**	**0.22**	**0.10**	0.04
S103	**0.73**	**0.41**	**0.15**	0.05
S104	**0.39**	**0.40**	**0.49**	0.03
S106	**0.85**	**0.81**	0.23	0.21
S107	**0.67**	**0.30**	**0.27**	0.05
S108	**1.16**	**0.30**	**0.16**	0.05
S109	**0.55**	**0.17**	**0.11**	0.05
S110	**0.64**	**0.55**	**0.30**	0.05
S111	**0.75**	**0.46**	**1.31**	0.05
S112	**0.13**	**0.11**	**0.17**	0.04
S113	**1.52**	**0.53**	**0.34**	0.07
S114	**0.19**	**0.20**	**0.12**	0.05
S115	**1.66**	**1.76**	**1.90**	0.04
S116	**1.15**	**0.37**	**0.61**	0.07
S117	**2.07**	**1.90**	**2.20**	0.18

^Pa P^Serum samples from patients with pneumococcal pneumonia (PP1 and PP2) and from healthy adults (S49 to S117) were analyzed by IgG Rec-ELISA with individual recombinant proteins.

^Pb P^The cutoff values were determined for each serum as two times the OD readings obtained with the GST. Boldface type, OD values greater than the cutoff values.

**Figure 4:**
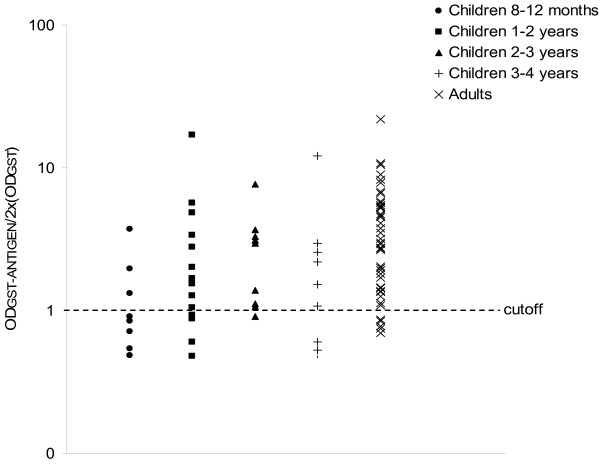
**Analysis of the B cell reactivity of the epiA peptide in healthy adults and children**. Immunoreactivity of the recombinant GST-epiA protein analyzed by IgG Rec-ELISA with serum samples from 48 blood donors and 40 children is shown. The values were calculated as the ratio of the OD readings obtained with the GST-epiA to twice the corresponding OD readings obtained with the GST.

Because of the broad reactivity of the epiA fragment, its recognition by antibodies present in sera from young children was also investigated. Serum samples from forty children with an age comprised between 1 and 4 years were assayed with the recombinant GST-epiA antigen by Rec-ELISA. As shown in Fig. 4, specific IgG antibodies against the epiA polypeptide were detected in sera from the majority of subjects (27 out of 40), thus demonstrating that 68% of children less than 4 years old had specific antibodies against this short polypeptide.

Next, a detailed epitope mapping was performed employing affinity-purified antibodies. Specific immunoglobulins against the GST-epiA fusion protein were purified from 5 epiA-reactive sera and then assayed with the recombinant GST-epiA, GST-epiB and GST-SP4 antigens by Rec-ELISA. As a negative control, the IgG reactivity against wild-type GST protein was also assessed. As shown in Table 2, the affinity-purified anti-epiA antibodies specifically recognized both the epiA and epiB polypeptides as well as the large SP4 fragment, indicating that a common epitope was present in the epiA and epiB protein sequences.

**Table 2:** Cross-reactivity of epiA and epiB polypeptides with affinity-purified anti-epiA antibodies P^aP^

**Antibody source**	**IgG Rec-ELISA (OD) **P^bP^			
	
	**GST-epiA**	**GST-epiB**	**GST-SP4**	**GST**
Sera mixture	**2.802**	**1.332**	**2.866**	0.256
Anti-epiA (150 ng mL^-1^)	**2.107**	**2.124**	**2.274**	0.092
Anti-epiA (30 ng mL^-1^)	**0.601**	**0.609**	**0.660**	0.047

^Pa P^Recombinant antigens were P^P^analyzed by IgG Rec-ELISA with a homogeneos mixture of human sera (diluted 1:50) or with affinity-purified anti-epiA antibodies

^Pb P^The cutoff values for the IgG Rec-ELISA with the recombinant GST-epiA, GST-epiB, and GST-SP4 proteins were calculated as two times the OD readings obtained with the GST. Boldface type, OD values greater than the cutoff values.

**Figure 5:**
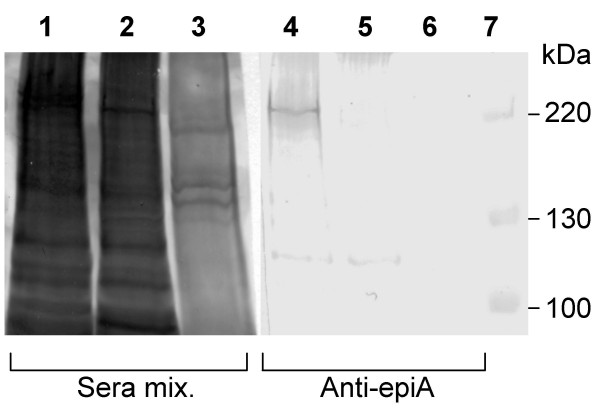
**Recognition of pneumococcal *iga *gene product by affinity-purified anti-epiA antibodies**. Western blot analysis on whole cell lysates from *S. pneumoniae *R6 (lanes 1 and 4) and FP174 strains (lanes 2 and 5), and from *S. gordonii *(lanes 3 and 6). A homogeneous mixture of 5 human sera (lanes 1–3) or affinity-purified anti-epiA antibodies (lanes 3–6) were used.

Next, to exclude non-specific immunoreactivity due either to altered conformation of the small polypeptides with respect to native IgA1 protease or to cross-reactivity of unrelated antibodies in human sera, a Western blot analysis was performed. To this aim, a whole cell lysate of *S. pneumoniae *was challenged with the affinity-purified anti-epiA antibodies. Bacterial lysates from pneumococcal *iga*-deficient mutant strain FP174 and from *Streptococcus gordonii *were assayed as controls. As shown in Figure 5, a unique protein band with an apparent molecular mass higher than 220 kDa specifically reacted with the anti-epiA antibodies in an unencapsulated strain of pneumococcus (R6). In the FP174 mutant strain, in which the *iga *gene was deleted, as well as in *S. gordonii*, such a protein band was not visible, demonstrating that the anti-epiA antibodies specifically recognized the pneumococcal enzyme.

Finally, to investigate whether the epiA polypeptide is exposed on the bacterium and is recognizable by anti-epiA antibodies, a cell-ELISA analysis was performed on whole *S. pneumoniae *cells. The FP174 strain was used as the negative control. The affinity-purified anti-epiA antibodies reacted with intact bacterial cells (strain R6) but not with the *iga*-deleted FP174 strain (data not shown), indicating that the epiA epitope within the tandem repeats of the pneumococcal enzyme is indeed exposed in the native state. Noteworthy, our results are in close agreement with a previous work where it was demonstrated that the tandem repeats of *S. sanguis *IgA1 protease contain immunodominant epitopes exposed in the denatured and native state [16].

## Discussion

Bacterial IgA1 proteases are highly specific endopeptidases that are secreted by a small number of bacteria associated with humans [23,24]. IgA1 protease-producing bacteria include the mucosal pathogens *N. meningitidis*, *N. gonorrhoeae*, *H. influenzae*, *S. pneumoniae*, *U. urealyticum *and some members of pharyngeal microfloras such as *Prevotella*, *Gemella haemolysans*, *S. sanguis*, *S. oralis *and *S. mitis *[14,22]. The importance of this bacterial enzyme in the interaction with the host is evidenced by the fact that most of these enzymes are specific for human IgA only, and that their impact on the host is so great as to become one of the few recognised factors of positive evolutionary selection on the human genome [25]. For the bacterial pathogens colonizing mucosal surfaces, IgA1 protease production represents a virulent factor and contributes to the pathogenesis of invasive infection by cleaving the Fc receptor from secreted immunoglobulins A1, thus preventing opsonophagocytosis [5]. IgA1 proteases have been shown to be targets of enzyme-neutralizing antibodies in serum and secretions [26], which may be induced in a state of bacterial carriage as well as during invasive infection [27].

We previously demonstrated the potential of phage-display technology in identifying B-cell epitopes recognized by the human immune response against *Toxoplasma gondii *and *S. pneumoniae *infection [20,28-30], even though such a technique usually favors the identification of linear epitopes whereas conformational epitopes are often overlooked. Nevertheless, the present work allowed the identification of antigenic regions of the pneumococcal IgA1 protease that are broadly recognized by human antibodies. Challenging a lambda-display library of *S. pneumoniae *with sera from patients hospitalized for pneumococcal pneumonia enabled the selection of three distinct clones harboring a common region of the IgA1 protease. All clone sequences matched the pneumococcal enzyme encompassing amino acids 360–590. The immunoreactivity of the selected fragments, expressed as GST fusion products, was assessed; overall, IgG antibodies in serum samples from more than 90% of healthy adults reacted with at least one antigen fragment, which emphasizes the broad recognition of pneumococcal IgA1 protease by the human B cell response.

Because of the broad reactivity of the selected IgA1 protease fragments, a detailed characterization of their common region, composed by 72 amino acids, was performed. This lead to classify the sequence corresponding to residues 420–457 (epiA peptide) of the *iga *gene product as the most reactive polypeptide, which was recognized by antibodies present in sera from 90% of healthy adults and 68% of children with an age comprised between 1 and 4 years, thus suggesting that the human antibody response against IgA1 protease occurs during childhood. Noteworthy, our data are in agreement with a previous study showing that IgG antibodies from the majority of children less than two years old specifically reacted with pneumococcal antigens, including the IgA1 protease [19].

The evidence that affinity-purified anti-epiA antibodies also reacted with the epiB fragment (residues 458–493) indicated that a common epitope within polypeptides was recognized by human sera. Notably, a repeated 9-mer sequence VVTDKGEPE is present twice in epiA and once in epiB fragments (Fig. 1 and 2). This duplication might account for the larger reactivity of the epiA peptide with respect to the epiB peptide.

The IgA1 protease of *S. pneumoniae *is a major antigen expressed in all known pneumococcal strains [7]. Despite the pneumococcal enzyme has a peculiar structure among bacterial proteases, homology searches have indicated that the *iga *gene of *S. pneumoniae *is also found in other streptococci and related organisms [8,14,15,22]. As already noted by others, the repeats region shows high conservation within the different pneumococcal strains and between *S. pneumoniae *and the most closely related *S. mitis *(90% identity), while homology to IgA proteases from other streptococci is significantly lower in this segment when compared to the rest of the molecule [8,22]. The epiA peptide identified in this work is conserved in all pneumococci and in two out of three *S. mitis *strains [22], while it is not conserved in other oral streptococci sequenced so far. The fact that the domain covered by epiA is not shared with oral streptococci other than *S. mitis *suggests that this domain is probably not interchangeable, which reduces the risk of escape mutants by horizontal gene transfer. In other words, this would indicate that any reservoir of divergent IgA1 proteases in non pneumococcal species should not be imported readily by recombination into pneumococcus.

We have shown here that an immunodominant polypeptide (epiA peptide), located within the sequence repeats domain of pneumococcal IgA1 protease, is well conserved among several serotypes and strains of *S. pneumoniae *but not to the corresponding regions of the *iga *gene products from other streptococci such as *S. oralis*, *S. sanguis *and *S. sanguinis*. However, we also found a very high sequence homology of this protein region between *S. pneumoniae *and *S. mitis *(see Fig. 2), suggesting that the observed high rate of seropositivity against pneumococcal IgA1 protease might be due to early colonization with bacteria species other than pneumococcus.

## Conclusion

The broad and specific recognition of the epiA polypeptide by human sera demonstrates that the pneumococcal IgA1 protease contains an immunodominant B-cell epitope. In perspective, it will be of great interest to investigate the ability of the anti-epiA antibodies in conferring protective immunity against *S. pneumoniae *infection in animal models. The use of phage display libraries to identify microbe or disease-specific antigens recognized by the human sera is a promising and valuable approach to epitope discovery.

## Methods

### Bacterial strains

Bacteria were grown in tryptic soy broth (TSB) in a 5% COB_2B_-enriched atmosphere at 37°C. Where necessary, streptomycin and kanamycin were used at a final concentration of 500 μg mLP^-1P^. *Streptococcus pneumoniae *R6 [31], FP174 (ΔSP1154/*iga*) [12] and *S. gordonii *[32] strains were used for this study.

### Affinity selection of the *S. pneumoniae *display library and serum samples

Serum samples from two patients with a respective age of 2 and 64 years old, collected during patient's hospitalization for acute pneumococcal pneumonia, were used for the affinity selection of a whole genome lambda-display library of *S. pneumoniae *D39 strain [20]. The construction of the pneumococcal library and the selection of the library with human sera were performed as previously described [33]. Briefly, magnetic beads linked to Protein G (Dynal, Norway) were incubated for 40 min at room temperature with 10 μL of serum. Beads were then incubated with 5 × 10^10 ^plaque forming units (PFU) of the phage library (complexity of the library, 2 × 10^7 ^independent clones) for 3 hours at room temperature. *Escherichia coli *cells were infected with phage pools by mixing the beads and the bacterial cells, and recombinant phages were amplified by using standard procedures [34]. After two rounds of affinity selection, single phage clones were isolated from phage pools by immunoscreening [28].

Forty-eight serum samples collected from 48 blood donors were anonymously collected and then used for the analysis of selected antigen fragments' immunoreactivity. Forty sera from 40 children born to mothers with congenital infections in pregnancy (i.e. *Toxoplasma gondii *and human cytomegalovirus infection) and referred for post-natal follow up of congenital diseases were also included in the study. Sera were collected from children with asymptomatic diseases with an age comprised between 1 and 4 years, and the serum samples were assayed in a blinded fashion.

### Cloning and recombinant protein expression and purification

DNA inserts of phage clones SP2 and SP4 were amplified by PCR, digested with *Spe I *and *Not I *endonucleases and subcloned into the bacterial vector pGEX-SN [21]. DNA fragments encoding for amino acids 420–457 and amino acids 458–493 of the *S. pneumoniae *IgA1 protease (GenBank Q54875) were PCR amplified from phage clone SP4 by using oligonucleotides K708 (5'-ACGACTAGTGCAATTCAGCCTGAGT TGCCC-3') and K709 (5'-GGTGCGGCCGCTCACTCTGGTTGAACCTCAGTCTC-3'), and oligonucleotides K710 (5'-CGAACTAGT TCGCCAGATACTGTGGTAAG-3') and K711 (5'-GGTGCGGCCGCTCACTTCTCAACCGGAGTTTCAC-3'), respectively. PCR products were digested with *Spe I *and *Not I *enzymes and then cloned into pGEX-SN plasmid. Competent *Escherichia coli *cells (AD202 strain) [34] were transformed with recombinant plasmids and single clones were isolated.

Recombinant proteins produced in *E. coli *as fusion proteins with glutathione S-transferase (GST) were purified from the cytoplasm of bacterial cells by affinity chromatography, as previously described [29]. Briefly, recombinant *E. coli *was induced with isopropyl-thiogalactopyranoside (IPTG), centrifuged and suspended in 10 mM Tris-HCl (pH 8), 150 mM NaCl, 100 μg mL^-1 ^of lysozyme and protease inhibitors (Boheringer, Germany). The mixture was sonicated, and Triton-X100 was added to a final concentration of 1%. After centrifugation at 10,000 × g for 30 min at 4°C, the supernatant was incubated with Glutathione-Sepharose (Amersham-Pharmacia Biotech, Sweden) and GST-proteins were eluted by following the manufacturer's instructions. Finally, protein purity and content were assessed by sodium dodecyl sulfate-polyacrilamide gel electrophoresis (SDS-PAGE) and by Bradford assays, respectively.

### Recombinant protein enzyme-linked immunoassays (Rec-ELISA)

Maxisorb multiwell plates (Nunc) were adsorbed with recombinant proteins (GST-SP2, GST-SP4, GST-epiA, GST-epiB and GST) at a concentration of 5 μg mL^-1 ^in 50 mM NaHCOB_3B_, pH 9.6. After incubation overnight at 4°C, the plates were blocked with 5% non-fat dry milk and 0.05% Tween-20 in PBS (blocking buffer) and incubated for 1 h at 37°C with serum samples diluted 1:50 in blocking buffer. The plates were washed with 0.05% Tween-20 in PBS, and anti-human IgG horseradish peroxidase-conjugated antibodies (Sigma-Aldrich, USA) were then added to each well. Finally, incubating the plates with the chromogenic substrate tetramethylbenzidine (TMB; Sigma Aldrich) revealed the enzymatic activity. The results were recorded as the difference between the absorbance (optical density, OD) at 450 and at 620 nm, as detected with an automated ELISA reader (Labsystem Multiskan, Finland). Assays were performed in duplicate, and average values were calculated. GST carrier protein was used as the negative control in every assay.

### Purification of anti-epiA antibodies from human sera

The GST-epiA fusion protein was immobilized to a NHS-activated-agarose column (Amersham-Pharmacia Biotech, Sweden) in accordance with the manufacturer's instructions. A mixture of sera from five blood donors (sera S90, S117, S115, S62 and S76) were allowed to flow through the column overnight to permit the binding of specific antibodies to the recombinant protein. After extensively washing the column with PBS and then with a solution containing 0.5 M NaCl in PBS, the antigen-specific antibodies were eluted with a solution of 50 mM glycine (pH 3) containing 100 mM NaCl. The antibody concentration was determined by Bradford assay.

### Western blot analysis

*S. pneumoniae *strains R6 and FP174 and *S. gordonii *strain Challis [32] were grown in 15 mL of Tryptic Soy Broth (TSB, BD Biosciences) in standard conditions. Cells were collected by centrifugation at 3000 g for 15 min at 4°C and washed with PBS. Bacteria were suspended in a final volume of 0.5 mL of loading buffer (10 mM Tris-HCl, 1 mM EDTA, 1% SDS, 10 mM dithiothreitol, 10% glycerol, 0.01% bromophenol blue), heated for 15 min at 95°C and then subjected to SDS-PAGE. Proteins were transferred onto nitrocellulose membranes (BioTrace NT, Pure Nitrocellulose Blotting Membrane, Pall Life Science), which were subsequently blocked with 5% non-fat dry milk and 0.05% Tween-20 in PBS (blocking buffer). The filters were incubated overnight at 4°C with the affinity-purified anti-epiA antibodies used at a concentration of 150 ng mL^-1^. After being extensively washed with 0.05% Tween-20 in PBS, filters were incubated with anti-human IgG alkaline phosphatase-conjugated antibodies (Sigma-Aldrich, USA). Finally, protein bands were visualized by using nitroblue tetrazolium (NBT; Sigma Aldrich) and 5-bromo-4-chloro-3-indosyl phosphate (BCIP; Sigma Aldrich) as choromogenic substrates.

## Authors' contributions

FDP carried out affinity selection of the phage display library, purification of recombinant proteins and Rec-ELISA analysis. EB performed bacterial growth and Western blot analysis. AS performed the characterization of anti-epiA antibodies purified from human sera. FM provided clinical samples of pneumococcal pneumonia. MRO provided bio-informatics support and continuous help and discussions during the conduct of the work. FF provided helpful discussion and critical reading of the manuscript. NG designed the study and wrote the manuscript. All authors read and approved the final manuscript.
